# P018: A prospective study of catheter associated urinary tract infections and rationalisation of antibiotic use in a tertiary care centre in North India

**DOI:** 10.1186/2047-2994-2-S1-P18

**Published:** 2013-06-20

**Authors:** N Taneja, S Appanwar, M Biswal, B Mohan, MM Aggarwal, R M, AK Mandal

**Affiliations:** 1 Medical Microbiology, Chandigarh, India; 2 AUC, PGIMER, Chandigarh, India

## Introduction

Catheter associated bacteriuria is very common and there is a need to differentiate symptomatic CA-UTI from asymptomatic bacteriuria (CA-ASB) to rationalise antibiotic usage.

## Objectives

Aim was to evaluate prevalence of catheter CA-ABU v/s CA-UTI and to assess the antibiotic usage in CA-ABU and CA-UTI group.

## Methods

A prospective cohort longitudinal study was conducted by recruiting seventy consecutive patients with catheter in situ over a period of three months. Patients were categorized as symptomatic and asymptomatic CAUTI based on the CDC definition. Demographic profile, primary & other co-morbidities, type of catheter, indication and duration of catheterization, details of surgical procedure performed, antibiotic prophylaxis and therapy were noted. The bed occupancy rate, device utilization rates, total device days and device associated infections (DAI) rate were calculated using standard definitions. Microbiological data were noted and analysed. Data analysed using SPSS-17.

## Results

Out of 70 patients, 52 had bacteriuria of which 10 were symptomatic. Ratio of urinary catheter use was 0.69. Catheter utilisation rate was 40.29/1000 device days and the rate of CA-UTI was 12.8 /1000 device days. Median duration of hospitalisation was 29 and 30 days for CA-ASB and CA-UTI. There was no statistically significant difference between median duration of catheterisation (p value>0.5). Overall 737 DDDS were given. Though symptomatic patients received more antibiotics the difference from asymptomatic bacteriuria was not significant. Inappropriate antibiotics usage was noted in the form wrong class, 2 antibiotics of same class and wrong indication in many cases.

## Conclusion

Pilot study helped us to rationalize the antibiotic usage in our centre.

## Disclosure of interest

None declared

